# Supplementary material for: Impaired lysosomal acidification triggers iron deficiency and inflammation in vivo
Source: eLife. 2019 Dec 3;8:e51031. doi: 10.7554/eLife.51031 (PMC6917501; doi:10.7554/eLife.51031)
Supplement: Supplementary file 5. [file elife-51031-supp5.docx]

Supplementary file 5 List of mouse oligonucleotide sequences (qPCR primers and siRNA constructs)

| Oligonucleotide | SOURCE | IDENTIFIER |
| --- | --- | --- |
| HPRT qPCR  5’– CCTCCTCAGACCGCTTTTT – 3’  3’– AACCTGGTTCATCATCGCTAA – 5’ | This paper | N/A |
| RPS12 qPCR  5’– GAAGCTGCCAAGGCCTTAGA – 3’  3’– AACTGCAACCAACCACCTTC – 5’ | This paper | N/A |
| GAPDH qPCR  5’– TGTGTCCGTCGTTCTGA – 3’  3’– CCTGCTTCACCACCTTCTTGA – 5’ | This paper | N/A |
| TFRC qPCR  5’– GTTTCTGCCAGCCCCTTATTAT – 3’  3’– GCAAGGAAAGGATATGCAGCA – 5’ | This paper | N/A |
| FTH1 qPCR  5’– CAAGTGCGCCAGAACTACCA – 3’  3’– ACAGATAGACGTAGGAGGCATAC – 5’ | This paper | N/A |
| NDUFS3 qPCR  5’– TGGCAGCACGTAAGAAGGG – 3’  3’– CTTGGGTAAGATTTCAGCCACAT – 5’ | This paper | N/A |
| NDUFB2 qPCR  5’– CCCCGGTACAGGGAGTTTC – 3’  3’– GCCAAAATCGCCAAAGAATCCA – 5’ | This paper | N/A |
| SDHA qPCR  5’– GGAACACTCCAAAAACAGACCT – 3’  3’– CCACCACTGGGTATTGAGTAGAA – 5’ | This paper | N/A |
| UQCRC2 qPCR  5’– AAAGTTGCCCCGAAGGTTAAA – 3’  3’– GAGCATAGTTTTCCAGAGAAGCA – 5’ | This paper | N/A |
| COX10 qPCR  5’– AGAAGAGCTATACAGGGATTGCC – 3’  3’– CTGTGTGACATACATGCGCTT – 5’ | This paper | N/A |
| ATP5D qPCR  5’– CCACACTACAGGTCCTACGG – 3’  3’– CACAGAGGAGTCGGCATTCA – 5’ | This paper | N/A |
| TLR9 qPCR  5’– ACAACTCTGACTTCGTCCACC – 3’  3’– TCTGGGCTCAATGGTCATGTG – 5’ | This paper | N/A |
| MYD88 qPCR  5’– AGGACAAACGCCGGAACTTTT – 3’  3’– GCCGATAGTCTGTCTGTTCTAGT – 5’ | This paper | N/A |
| IRF7 qPCR  5’– GCGTACCCTGGAAGCATTTC – 3’  3’– GCACAGCGGAAGTTGGTCT – 5’ | This paper | N/A |
| STAT1 qPCR  5’– TCACAGTGGTTCGAGCTTCAG – 3’  3’– CGAGACATCATAGGCAGCGTG – 5’ | This paper | N/A |
| STAT2 qPCR 5’– GTTACACCAGGTCTACTCACAGA – 3’  3’– TGGTCTTCAATCCAGGTAGCC – 5’ | This paper | N/A |
| CXCL10 qPCR  5’– CCAAGTGCTGCCGTCATTTTC – 3’  3’– GGCTCGCAGGGATGATTTCAA – 5’ | This paper | N/A |
|  |  |  |
| continued |  |  |
| ISG15 qPCR  5’– GGTGTCCGTGACTAACTCCAT – 3’  3’– CTGTACCACTAGCATCACTGTG – 5’ | This paper | N/A |
| IFIT1 qPCR  5’– GCCTATCGCCAAGATTTAGATGA – 3’  3’– TTCTGGATTTAACCGGACAGC – 5’ | This paper | N/A |
| IFIT3 qPCR  5’– CCTACATAAAGCACCTAGATGGC – 3’  3’– ATGTGATAGTAGATCCAGGCGT – 5’ | This paper | N/A |
| IFI44 qPCR  5’– ATGCTCCAACTGACTGCTCG – 3’  3’– ACAGCAATGCCTCTTGTCTTT – 5’ | This paper | N/A |
| POLG qPCR  5’– GAGCCTGCCTTACTTGGAGG – 3’  3’– GGCTGCACCAGGAATACCAG – 5’ | This paper | N/A |
| TFAM qPCR  5’– GCTCTACACGCCCCTGGTTTCTGG –3’  3’– TCGCTGTAGTGCCTGCTGCTCCTG – 5’ | This paper | N/A |
| TFB2M qCPR  5’– TATAGAGCCGTTGCCTGATTCT – 3’  3’– GCCGCTTTCTTACATGCTATGTG – 5’ | This paper | N/A |
| POLRMT qPCR  5’– AGAAGGCTCCAGTAATGTCCA – 3’  3’– CCTGCATCAGTATGCTCACAA – 5’ | This paper | N/A |
| ENDOG qCPR  5’– TTCCGCGAGGATGACTCTGT – 3’  3’– CACCTGAGGCGCTACGTTG – 5’ | This paper | N/A |
| SSBP1 qPCR  5’– TTCAGTTACTTGGACGAGTAGGT – 3’  3’– CGCCACATCTCATTTGTTGCTA – 5’ | This paper | N/A |
| mtDNA qPCR  5’– CCTATCACCCTTGCCATCAT – 3’  3’– GAGGCTGTTGCTTGTGTGAC – 5’ | This paper | N/A |
| nDNA qPCR  5’– ATGGAAAGCCTGCCATCATG – 3’  3’– TCCTTGTTGTTCAGCATCAC – 5’ | This paper | N/A |
| TNFα qPCR  5’– CAGGCGGTGCCTATGTCTC – 3’  3’– CGATCACCCCGAAGTTCAGTAG – 5’ | This paper | N/A |
| PPARG qPCR  5’– GGAAGACCACTCGCATTCCTT – 3’  3’– GTAATCAGCAACCATTGGGTCA – 5’ | This paper | N/A |
| ANGPTL4 qPCR  5’– GCATCCTGGGACGAGATGAAC – 3’  3’– CCCTGACAAGCGTTACCACA – 5’ | This paper | N/A |
| PDK1 qPCR  5’–GGCGGCTTTGTGATTTGTAT –3’  3’– ACCTAGATCGGGGGATAAAC – 5’ | This paper | N/A |
| VEGFA qPCR  5’– CACCATGCCAAGTGGTCC – 3’  3’– TCTCAATCGGACGGCAGTA– 5’ | This paper | N/A |
| PHD3 qPCR  5’ – GGCCGCTGTACTGTAT – 3’  3’ – TTCTGCCCTTCAGCAT – 5’ | This paper | N/A |
| ATP6V1H qPCR  5’– CCAAGATGGACATTCGAGGTG – 3’  3’– CACTTTGTTGGCACGAACTTC – 5’ | This paper | N/A |
| SLC11A2 qPCR  5’– TTTAGCTTTCGTAAACTCTGGGC – 3’  3’– GTTTCCTGGGTCTAGGTAGGC – 5’ | This paper | N/A |
| MCOLN1 qPCR  5’– GCTGGGTTACTCTGATGGGTC – 3’  3’– CCACCACGGACATAGGCATAC – 5’ | This paper | N/A |
| SLC11A2-siRNA  mm.Ri.Slc11a2.13.1  5’– CAAUCAUUGGUUCUGACAUGCAGGA – 3’  3’–CCGUUAGUAACCAAGACUGUACGUCCU– 5’  mm.Ri.Slc11a2.13.3  5’– AGUACUUCUUCAUCCGAGUCCUGCA – 3’  3’–UAUUCAUGAAGAAGUAGCUCAGGACGU – 5’ | This paper | N/A |
| ATP6V1H-siRNA  mm.Ri.Atp6v1h.13.2  5’– AUUCCGGAAUUUCUUAGAAAAAUCA – 3’  3’–CGUAAGGCCUUAAAGAAUCUUUUUAGU – 5’  mm.Ri.Atp6v1h.13.3  5’– GAAAUAUUACUGUUACUUAUAUUCT – 3’  3’– CUCUUUAUAAUGACAAUGAAUAUAAGA – 5’ | This paper | N/A |
|  |  |  |
