# Supplementary material for: Impaired lysosomal acidification triggers iron deficiency and inflammation in vivo
Source: eLife. 2019 Dec 3;8:e51031. doi: 10.7554/eLife.51031 (PMC6917501; doi:10.7554/eLife.51031)
Supplement: Supplementary file 6. [file elife-51031-supp6.docx]

Antibody validation

Anti-FTH1 validated by siRNA in Alvarez et al (2017) Nature 551(7682): 639-643

Anti-TOM20 validated by siRNA in Wei et al (2015) J Virol 89(7): 3804-18.

Anti-GAPDH validated by knockdowns in Pasquier et al (2019) Nat Commun 10(1):3312.

Anti-GFAP Lindqvist R et al ( 2016) J Neuroinflammation 13:277.

Anti-HIF1a validated in this manuscript by an increased band density in the corresponding size after CoCl2 treatment in cells. Also validated by siRNA in Guo et al (2019) Nat Commun 10(1): 278.

Anti-LC3B manufacturer’s validation by siRNA

Anti-OXPHOS cocktail Antibody validated by manufacture. Also validated in Yonesiro et al (2019) Nature 572(7771):614-619.

Anti-SQSTM1 validated by siRNA in Wang et al (2019) Autophagy 13(2): 404-422.

Anti-VHL validated by siRNA in Ordonez-Navadijo et al (2015) Oncogene 35(5): 587-594.

Anti-VDAC1 validated by siRNA in Yang G et al (2019) Oncol Lett 18(3): 2340-2347 and in Jian et al (2018) Cell Rep 23(10): 2989-3005.

Anti-TFAM Manufacturer validated for WB in human, mouse and rat samples and detects band at predicted molecular weight

Anti-TNFa -Manufacturer’s validation by positive control TPA-treated macrophages detects band at predicted molecular weight.

Anti- HPRT Manufacturer’s validation by knockout in HAP1 cells.

Anti- Cleaved CASP3 validated by knockdown and overexpression by Luo Yongting et al (2019) Nat Commun 10: 3551 and manufacturers validation for WB.

Anti-Cleaved PARP manufacturer’s validation for WB

ATP5B was self-raised and validated in human mitochondria samples, detecting bands at predicted molecular size that were absent in the pre-immune serum. It has been verified and used in multiple publications like Aich et al (2018) eLife and in, Richter-Dennerlein et al (2016) Cell 167(2): 471-483.

COX1 was self-raised and validated in human mitochondria samples, detecting bands at predicted molecular size that were absent in the pre-immune serum. In addition, Ab was validated in mitochondria derived from Rho0 cells (lacking mtDNA). It has been used in multiple publications like Aich et al (2018) eLife and in, Richter-Dennerlein et al (2016) Cell 167(2): 471-483.

Anti-PLP and anti-MBP validated in induced conditional knockout mice in Luders et al (2019) Glia 67(4): 634-649 and in Luders et al (2017) Glia 65(11): 1762-1776.

Anti-FTL validated by overexpression and shRNA mediated silencing in Fan et al (2014) BBA Mol Cel Research 1843(11): 2775-2783.

Anti-Pex5 manufacturer’s enhanced antibody validation

Anti-ATP6V1H validated for WB by siRNA silencing in MEFs in this manuscript

Anti-VAPB- Validated for WB by manufacturer.

Anti-HA antibody validated by overexpression in this manuscript.

Anti-LAMP1 validated by overexpression in Conesa et al (2003) Biochem J 370(2): 703-711.
