# Supplementary material for: Impaired lysosomal acidification triggers iron deficiency and inflammation in vivo
Source: eLife. 2019 Dec 3;8:e51031. doi: 10.7554/eLife.51031 (PMC6917501; doi:10.7554/eLife.51031)
Supplement: Transparent reporting form [file elife-51031-transrepform.pdf]

1st Floor
24 Hills Road
Cambridge CB2 1JP, UK

P 01223 855340
W elifesciences.org
T @elife

eLife's transparent reporting form

We encourage authors to provide detailed information *within their submission* to facilitate the interpretation and replication of experiments. Authors can upload supporting documentation to indicate the use of appropriate reporting guidelines for health-related research (see EQUATOR Network), life science research (see the BioSharing Information Resource), or the ARRIVE guidelines for reporting work involving animal research. Where applicable, authors should refer to any relevant reporting standards documents in this form.

If you have any questions, please consult our Journal Policies and/or contact us: editorial@elifesciences.org.

Sample-size estimation

- You should state whether an appropriate sample size was computed when the study was being designed
- You should state the statistical method of sample size computation and any required assumptions
- If no explicit power analysis was used, you should describe how you decided what sample (replicate) size (number) to use

Please outline where this information can be found within the submission (e.g., sections or figure legends), or explain why this information doesn't apply to your submission:

No statistical method was used to compute sample sizes. Indeed power analyses was not performed to predetermine number of samples. Due to the consistency of differences between experimental conditions, sample sizes were deemed sufficient. Typically, the mean of technical replicates represented one biological sample, and there were at least three biological replicates for each experiment.

Replicates

- You should report how often each experiment was performed
- You should include a definition of biological versus technical replication
- The data obtained should be provided and sufficient information should be provided to indicate the number of independent biological and/or technical replicates
- If you encountered any outliers, you should describe how these were handled
- Criteria for exclusion/inclusion of data should be clearly stated
- High-throughput sequence data should be uploaded before submission, with a private link for reviewers provided (these are available from both GEO and ArrayExpress)

Please outline where this information can be found within the submission (e.g., sections or figure legends), or explain why this information doesn't apply to your submission:

Each biological replicate (at least 3 for each experiment) was made of the mean of technical replicates. The exact number of replicates is detailed in each figure legend. For example, for cell culture, each independent experimental replication was one biological sample. And for each independent experiment, at least two technical replicates were typically made. For experiments involving mice, each experimental replicate was carried out with different cohorts, and the number of different mice per genotype in all experiments represented the sample size for that genotype.

eLife Sciences Publications, Ltd is a limited liability non-profit non-stock corporation incorporated in the State of Delaware, USA, with company number 5030732, and is registered in the UK with company number FC030576 and branch number BR015634 at the address 1st Floor, 24 Hills Road, Cambridge CB2 1JP | August 2014

Statistical reporting

- Statistical analysis methods should be described and justified
- Raw data should be presented in figures whenever informative to do so (typically when N per group is less than 10)
- For each experiment, you should identify the statistical tests used, exact values of N, definitions of center, methods of multiple test correction, and dispersion and precision measures (e.g., mean, median, SD, SEM, confidence intervals; and, for the major substantive results, a measure of effect size (e.g., Pearson's r, Cohen's d)
- Report exact p-values wherever possible alongside the summary statistics and 95% confidence intervals. These should be reported for all key questions and not only when the p-value is less than 0.05.

Please outline where this information can be found within the submission (e.g., sections or figure legends), or explain why this information doesn't apply to your submission:

All statistical tests used in this study are described in the corresponding figure legends, as well as in detail in the methods. Data are typically summarized as mean±SEM.

(For large datasets, or papers with a very large number of statistical tests, you may upload a single table file with tests, Ns, etc., with reference to sections in the manuscript.)

Group allocation

- Indicate how samples were allocated into experimental groups (in the case of clinical studies, please specify allocation to treatment method); if randomization was used, please also state if restricted randomization was applied
- Indicate if masking was used during group allocation, data collection and/or data analysis

Please outline where this information can be found within the submission (e.g., sections or figure legends), or explain why this information doesn't apply to your submission:

No randomization was applied. Samples were allocated into biological groups: control or treated cells. Mouse samples (cells or tissues) were allocated into WT and KO. In addition, for Iron-enriched diet supplementation experiments, allocated biological groups were WT and KO, with or without iron-enriched chow. For almost all mouse experiments, age-and sex-matched littermate controls were used for each setup.

Additional data files ("source data")

- We encourage you to upload relevant additional data files, such as numerical data that are represented as a graph in a figure, or as a summary table
- Where provided, these should be in the most useful format, and they can be uploaded as "Source data" files linked to a main figure or table
- Include model definition files including the full list of parameters used
- Include code used for data analysis (e.g., R, MatLab)
- Avoid stating that data files are "available upon request"

Please indicate the figures or tables for which source data files have been provided:

The RNASeq data generated in this study from the Gaa WT and KO cortices is deposited in the Gene Expression Omnibus (GEO) repository with accession number GSE134704. Publicly available datasets used in this study were previously deposited in the GEO database, and are referenced appropriately. Primer sequences are uploaded as supplementary table 5.

eLife Sciences Publications, Ltd is a limited liability non-profit non-stock corporation incorporated in the State of Delaware, USA, with company number 5030732, and is registered in the UK with company number FC030576 and branch number BR015634 at the address 1st Floor, 24 Hills Road, Cambridge CB2 1JP | August 2014
